# Association between the confluent form of pancreatic and bile duct and histopathological findings in pancreaticobiliary maljunction: A case series study

**DOI:** 10.1016/j.amsu.2021.102180

**Published:** 2021-02-23

**Authors:** Masaki Horiike, Yoshiki Morotomi, Shigekazu Takemura, Shogo Tanaka, Hiroji Shinkawa, Shigeo Hashimoto, Kenichi Wakasa, Shoji Kubo

**Affiliations:** a Department of Pediatric Surgery, Japanese Red Cross Society Wakayama Medical Center, 4-20, Komatsubara-dori, Wakayama City, Japan; b Department of Pediatric Surgery, Osaka City University Graduate School of Medicine, 1-4-3 Asahimachi, Abeno-ku, Osaka, 545-8585, Japan; c Department of Pediatric Surgery, Kitano Hospital, The Tazuke kofukai Medical Research Institute, 2-4-20 Ohgimachi, Kita-ku, Osaka, 530-8480, Japan; d Department of Hepato-Biliary-Pancreatic Surgery, Osaka City University Graduate School of Medicine, 1-4-3 Asahimachi, Abeno-ku, Osaka, 545-8585, Japan; e Department of Pathology, PL Hospital, 2240 Shindo, Tondabayashi, 584-8585, Japan; f Department of Pathology, Ishikiriseiki Hospital, 18-28 Yayoicho, Higashi, Osaka, 579-8026, Japan

**Keywords:** Pancreaticobiliary maljunction, Confluent forms, Gallbladder carcinogenesis, Congenital biliary dilatation, Histopathological search

## Abstract

**Introduction:**

Pancreaticobiliary maljunction (PBM) is a congenital anomaly wherein the persistent reflux of the pancreatic juice into the biliary tract induces biliary tract cancer. The prediction criteria for gallbladder carcinogenesis have been reported previously through results obtained from examining carcinogenic and non-carcinogenic cases with the parameters that classified each confluent form in PBM. This study aimed to validate these previous study results and provide new recommendations for gallbladder carcinogenesis prevention.

**Methods:**

Twenty-four patients with PBM underwent hepaticojejunostomy. The prediction criteria for gallbladder carcinogenesis consist of three elements. The confluent forms that satisfied none or one of the three criteria were defined as a low score group, and those that satisfied two or three were defined as the high score group. Immunohistology and pathological search were performed on the gallbladders' sections in both groups to evaluate chronic inflammation.

**Results:**

The cases with dysplasia, positive Ki67 labeling index, and gallbladder cancer were more common in the high score group and tended to have more lymphocyte infiltration. These findings indicate that the degree of inflammation and cell proliferation might be more severe in the high score group than in the low score group.

**Conclusions:**

There is a close relationship between the confluent form and the histopathological findings of the gallbladder in patients with PBM. The confluent forms observed in the high score group might have an additional correlation with increased proliferation activity and subsequent malignant transformation of the gallbladder epithelium.

## Highlights

• Pancreaticobiliary maljunction (PBM) may induce biliary tract cancer.
• This is the first report showing a correlation between the form in PBM and pathological findings of the gallbladder.
• We were able to show that PBM has a group with confluent forms with a strong carcinogenic tendency.

## 1 Introduction

Pancreaticobiliary maljunction (PBM) is a congenital anomaly wherein the pancreatic and bile ducts merge outside the duodenal wall, and the bile and pancreatic juices flow into each other because the sphincter of Oddi does not control the junction [1]. The persistent reflux of pancreatic juice into the biliary tract causes chronic inflammation, with damage followed by healing in the biliary mucosal epithelia, which in conjunction with DNA mutations, promotes carcinogenesis [[1], [2], [3], [4], [5]], which in turn is the most important prognostic factor.

**Fig. 1:**
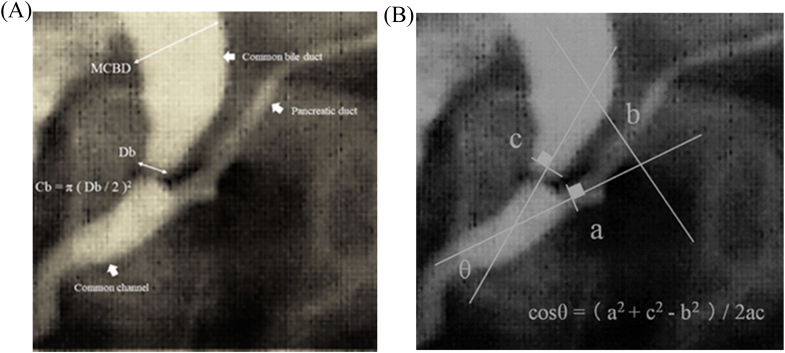
a: Visual illustration of θ, cos θ, and three side length of the triangle a, b, and c. The angle (θ) is formed from the lines extending from the pancreatic and bile ducts. The lengths of the triangle forming θ are defined as a and c, and the length of the intersection is defined as c. The trigonometric function cos θ was calculated as follows: cos θ=(a^2^+c^2^-b^2^)/2ac. b: Visual illustration of MCBD, Db, and Cb. MCBD, maximum diameter of the congenital biliary dilatation: Db, diameter of the biliary duct; Cb, bile duct cross-sectional area in the junction site. Cb was calculated as follows: π (Db/2)^2^.

A previous study [6] hypothesized that there might be differences in the quantity and duration of the pancreatic juice reflux into the biliary tract in each confluent form. These differences could be associated with the presence or absence of gallbladder carcinoma. The angle (ϴ) formed by the confluence of the bile and the pancreatic ducts (Fig. 1a), the bile duct cross-sectional area at the junction (Cb, Fig. 1b), and the ratio of the maximum diameter of the congenital biliary dilatation to the bile duct diameter at the junction site (MCBD/Db, Fig. 1c) were important parameters related to carcinogenesis of the gall bladder.

This study aimed to validate the previous study results by histopathological analyses in patients with PBM with or without gallbladder cancer and develop new predictive criteria for gallbladder cancer in patients with PBM.

## 2 Material and methods

### 2.1 Patients

The subjects were 24 patients with PBM who underwent hepaticojejunostomy between September 1990 and September 2017 at our hospital. Patients’ data, such as clear medical image findings, test results, and pathological specimens with no necrosis, breakage, or loss, were available. PBM was diagnosed according to the diagnostic criteria for PBM by the Japanese Study Group on PBM [7]. The age of the patients ranged from 9 days to 66 years.

We scored each confluent form in each patient using the following parameters: cos ϴ, Cb, and MCBD/Db, affecting gall bladder carcinogenesis [6]. The prediction criteria for gallbladder carcinogenesis were defined as follows: (1) cosϴ ≥0.84, (2) Cb ≥ 8.0, and (3) MCBD/Db ≤ 4.3. The series of confluent forms that satisfied none or only one of the three elements of the criteria was defined as the low score group, and the series of confluent forms that satisfied two or three was defined as the high score group.

### 2.2 Histopathology and immunohistochemistry

The specimens were fixed in 10% formaldehyde, paraffin-embedded, and cut into 4-μm serial sections. Hematoxylin-eosin and immunohistochemical staining were subsequently performed on the sections. Immunohistochemistry was performed using a two-step method. The sections were deparaffinized, immersed in citrate buffer (0.01 mol/L, pH 6.0), and placed in a microwave oven (98 °C) for 20 min. Endogenous peroxidase was blocked with 3% hydrogen peroxide/methanol. The sections were incubated overnight with a rabbit monoclonal antibody against Ki67 antigen (MIB-1) and a mouse monoclonal antibody against the p53 antigen (DO-7) as primary antibodies. After that, the slides were rinsed gently with phosphate-buffered saline, and Universal Kit (VENTANA ultra View Universal DAB Detection Kit) was used as the secondary antibody. Incubation with 3,3-diaminobenzidine tetrahydrochloride was performed for 10 min as a substrate chromogen solution to produce a brown color. Finally, the sections were counterstained with Mayer's hematoxylin.

### 2.3 Pathological parameters

We studied the histological features based on the findings of chronic inflammation in the mucosal epithelium of the gallbladder using the following parameters: flattening (or shortening) of the mucosal epithelium, lymphocyte infiltration, stromal fibrosis, presence of Rokitansky Ashoff sinus (RAS), papillary proliferation of the mucosal epithelium (hyperplasia), dysplastic change in the mucosal epithelium (dysplasia), gallbladder carcinoma, and immunohistochemical findings of Ki67 and p53. The results of Ki67 and p53 were evaluated in the most clearly stained areas of the non-cancerous epithelium, even in patients with gallbladder carcinoma. These pathological findings were examined and assessed by two skilled pathologists.

We used the Ki67 labeling index (LI) to assess the pathological specimens' proliferative activities with PBM. Manual scores of Ki67 were generated by independently counting at least three high-power fields (40× objective) of the most mitotically active area in each section (hot spot). Ki67 LI ≥ 10% was considered positive, whereas Ki67 LI<10% was considered a negative result.

Each cell of the gallbladder epithelium with brown-yellow granules in the nucleus was positive for immunostaining of p53, which is a tumor suppressor gene. We defined the criteria for positive staining of p53 in this study as follows: After counting positive cells in 10 high-power fields (×400) of all specimens, an expression rate of ≥5% was considered positive, whereas an expression rate of <5% was considered a negative result.

### 2.4 Ethics

This study was conducted in accordance with the Declaration of Helsinki and reported in line with the Strengthening the Reporting of Cohort Studies in Surgery (STROCSS) 2019 guidelines [8]. The study was approved by the Ethics Committee of our hospital (No.4013).

### 2.5 Theory/calculation

Continuous variables were examined using the Wilcoxon test, whereas categorical data were examined using the χ^2^ test or Fisher's exact test. Differences were considered significant at *p* < 0.05. Data were analyzed using JMP version 13 (SAS Institute Inc. Cary, NC, USA).

## 3 Results

### 3.1 Clinical characteristics

Of the 24 patients in this study, 19 were classified in the low score group, and 5 were included in the high score group. The age, sex, presence of biliary calculus, levels of hepatobiliary enzymes, bilirubin level, and white blood cell count were not significantly different between the groups. Comparing the confluent forms between both groups by Todani's classification, it was observed that the proportion of Ic type was significantly higher in the high score group than in the low score group. The results are summarized in Table 1.

**Table 1:** Clinical characteristics between low score group and high score group.

	low score (score 0–1)	high score (score 2–3)	
Variable	n = 19	n = 5	p value
Age	6 (0–55)	51 (3–57)	0.06
Male: female	1:4	6:13	0.61
Biliary calculus	7/12 (58%)	1/4 (25%)	0.47
AST U/L	34 (13–640)	32 (25–544)	0.55
ALT U/L	15 (8–331)	44 (9–682)	0.43
γGTP U/L	27 (7–1101)	138 (12–1104)	0.41
ALP U/L	573 (96–7800)	550 (174–2604)	0.59
T-Bil mg/dL	0.6 (0.3–16.2)	1.1 (0.5–3.9)	0.25
D-Bil mg/dL	0.2 (0.1–4.6)	0.2 (0.1–2.8)	0.68
LDH U/L	406 (165–1310)	285 (194–312)	0.28
WBC/μL	7400 (4600–24400)	7100 (4800–7400)	0.30

Todani Classification			
Іa	6	1	
Ic	4	4	
ⅣA	9	0	0.04

Of the 5 patients in the high score group, 4 had gallbladder cancer.

**Fig. 2:**
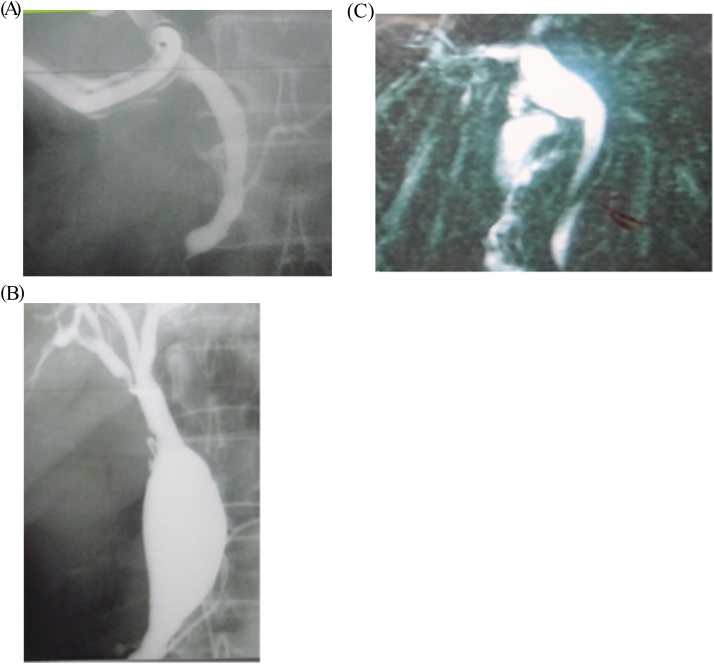
a–c: The images showing the type Ic confluence form of PBM patients with gallbladder carcinogenesis. PBM, pancreaticobiliary maljunction.

Three of the four patients with gallbladder cancer were classified as type Ic (Fig. 2a, b, and 2c). Of the 3 patients, 2 met all three prediction criteria for gallbladder carcinogenesis, whereas 1 met two of the three prediction criteria. Another patient with gallbladder cancer was classified as type Ia according to Todani's classification (Fig. 2d) and met two of the three prediction criteria. Therefore, all 4 patients fulfilled the criteria for inclusion in the high score group. The median age of the 4 patients with gallbladder cancer was 52.5 years (range: 37–57 years). The median age of the 20 patients with no gallbladder cancer was 4.5 years (range: 0–55 years).

### 3.2 Histopathological and immunohistochemical findings

**Fig. 3:**
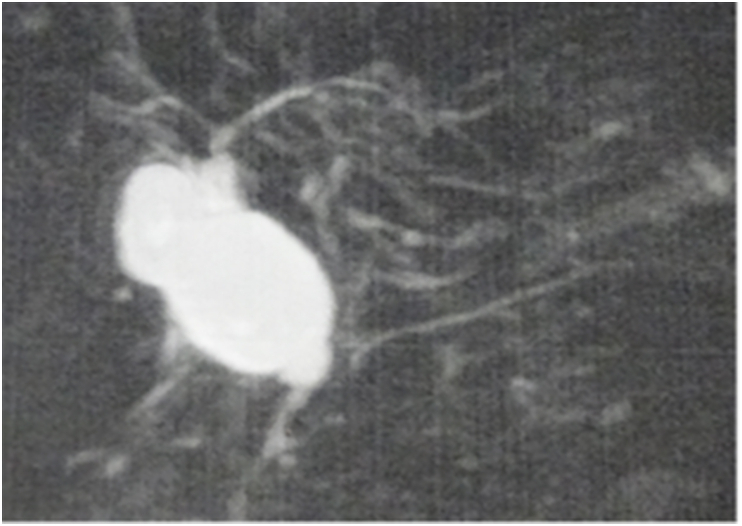
Type Ia confluence form of a patient with PBM with gallbladder cancer. PBM, pancreaticobiliary maljunction.

**Fig. 4:**
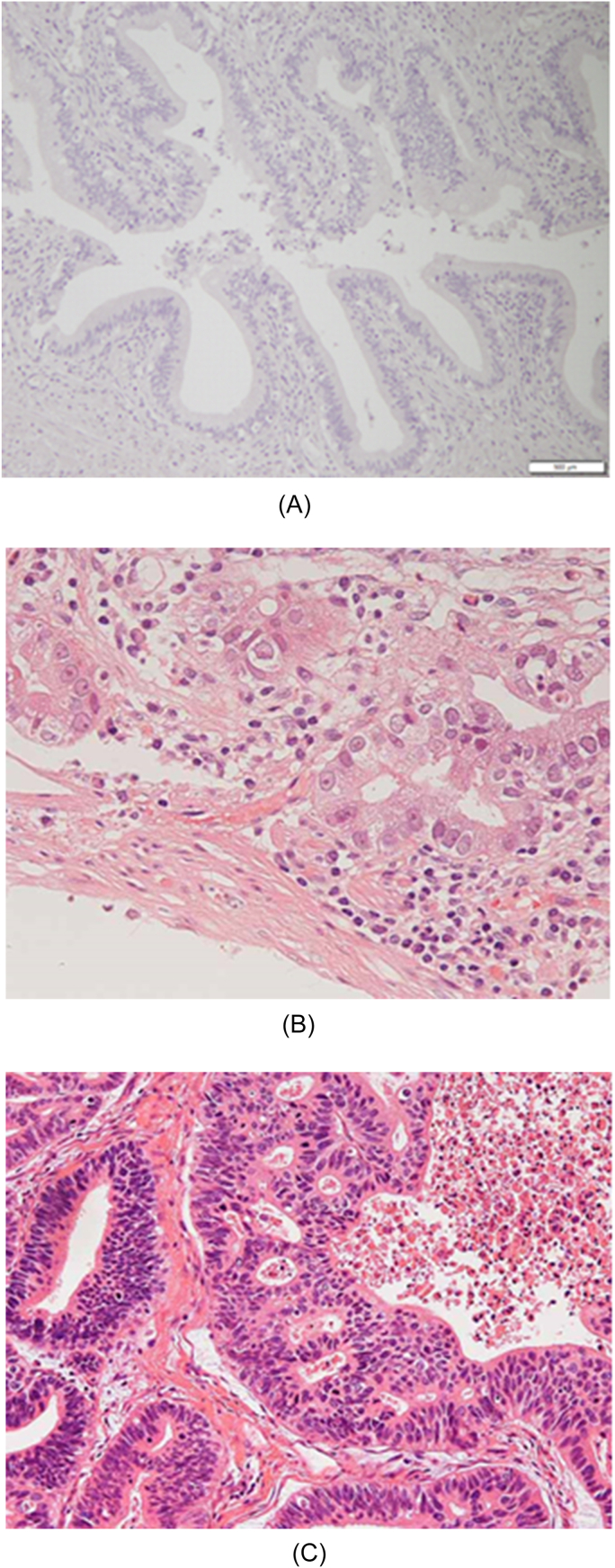
a: HE staining with a hyperplasia in PBM case (×100). b: HE staining with a dysplasia in PBM case (×200) c: HE staining with a gallbladder carcinoma in PBM case (×100). HE, hematoxylin-eosin; PBM, pancreaticobiliary maljunction.

**Fig. 5:**
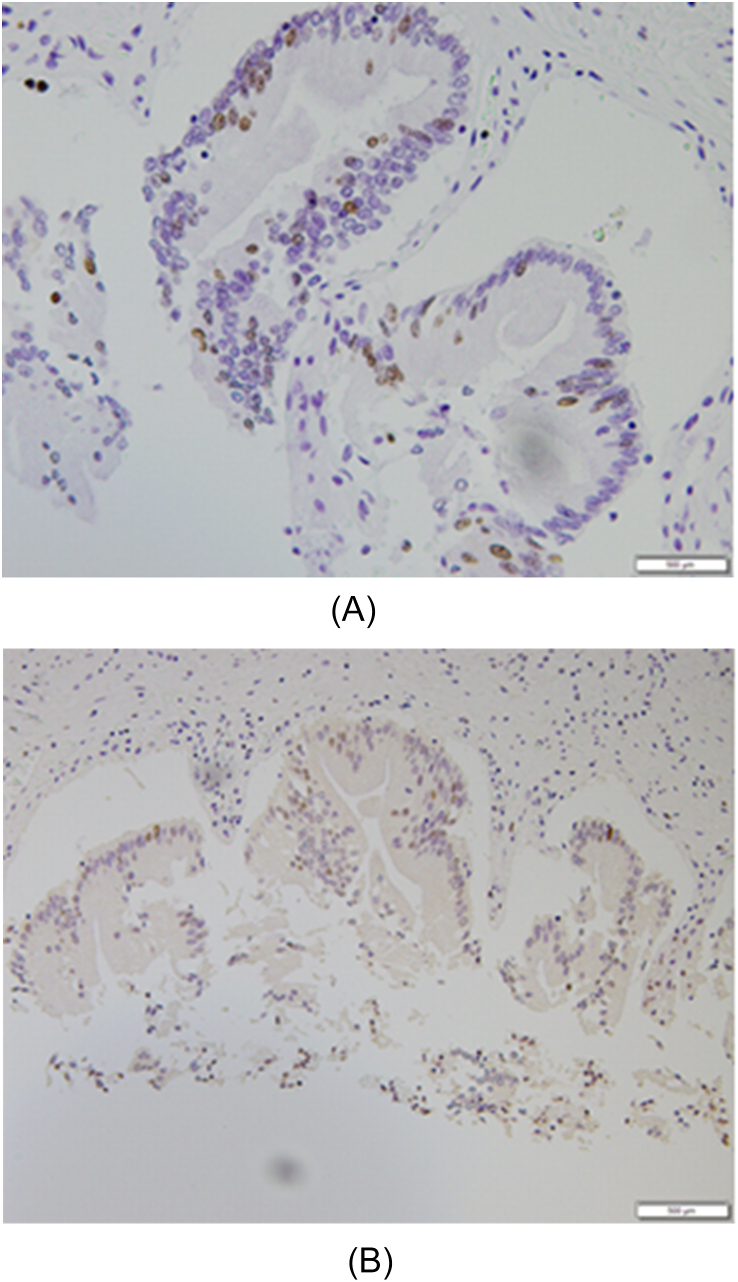
a: Ki 67 nuclear staining in non-cancerous lesion in PBM case. b: Immunostaining of p53 in non-cancerous lesion in PBM case. PBM, pancreaticobiliary maljunction.

Flattening of the mucosal epithelium was observed in 3, lymphocyte infiltration in 14, stromal fibrosis in 21, RAS in 10, hyperplasia (Fig. 3a) in 22, dysplasia (Fig. 4b) in 5, and gallbladder cancer (Fig. 4c) in 4 patients. Ki67 LI was positive in 5 patients (Fig. 5a), and positive expression of p53 was observed in 7 patients (Fig. 5b) (Table 2). Although the proportions of patients with flattening of the mucosal epithelium, lymphocyte infiltration, fibrosis of tissue stroma, RAS, and hyperplasia were not different between the groups, the proportion of patients with lymphocyte infiltration was higher in the high score group than in the low score group. Fibrosis of the tissue stroma was observed in a 1-year-old patient, lymphocyte infiltration was observed in a 2-year-old patient, and hyperplasia was observed in a 9-day-old patient. These findings indicate that chronic inflammation had started in the neonatal period regardless of the confluent form of PBM.

**Table 2:** The correlation between Score of confluent form and Histopathological and immunohistochemical parameters.

	total	low score group (score 0–1)	high score group (score 2–3)	p value
	n = 24	n = 19	n = 5	
Flattening (or shortening) of mucosal epithelium	3	2	1	0.57

Infiltration of lymphocyte	14	9	5	0.05

Fibrosis of tissue stroma	21	16	5	0.34

Rokitansky Aschoff Sinus	10	9	1	0.27

Papillary proliferation of mucosal epithelium (hyperplasia)	22	17	5	0.45

Dysplastic change of mucosal epithelium (dysplasia)	5	0	4	<0.05

Gallbladder carcinoma	4	0	4	<0.05

Ki67 labelling index (%)				
≧10	5	1	4	<0.05

p53 staining rate (%)				
≧5	7	5	2	0.55

Dysplasia was identified in 4 patients (80%) in the high score group, but not in the low score group. Ki67LI was positive in 4 patients (80%) in the high score group, but in only 1 patient (0.2%) in the low score group. The positive p53 expression was identified in 5 patients (26%) in the high score group and 2 (40%) in the low score group, and no significant differences were observed between the groups. Gallbladder cancer was found in 4 patients (80%) in the high score group, but not in the low score group. These findings indicate that the degree of inflammation might be more severe in the high score group.

## 4 Discussion

Carcinogenesis in PBM appears to be related to chronic inflammation resulting from the persistent reflux of pancreatic juice into the biliary tract. The hyperplasia-dysplasia-carcinoma sequence is regarded as the dominant mechanism underlying the development of biliary tract cancer in PBM. After Irwin and Morison reported the first case of carcinoma, which emerged from a congenital cyst [9], the correlation between congenital biliary dilatation and biliary cancer was widely recognized, and many studies have reported the relationship between bile duct dilatation and carcinogenesis in PBM [[10], [11], [12], [13], [14]]. However, to the best of our knowledge, there are no reports on biliary carcinogenesis focusing on the confluent form of the bile and pancreatic ducts. A previous case series study which included 37 patients with PBM who underwent hepaticojejunostomy and investigated the correlation between the confluent form and carcinogenesis hypothesized that the confluent form of PBM is related to gallbladder carcinogenesis [6]. This study confirms the correlation between the confluent forms of PBM and the pathological differences in the mucosal epithelium of the gallbladder.

The proportion of patients with signs of chronic inflammation, such as lymphocyte infiltration, stromal fibrosis, and hyperplasia, was considerably high (more than 66.7%) in patients with PBM. Funabiki et al. stated that the incidence of hyperplastic changes is significantly greater in the non-cancerous epithelia of the gallbladder of patients with PBM with or without cancer than in patients without PBM [15]. Seki et al. [16] and Espinoza JA et al. [17] reported that hyperplasia in PBM was an important precursor lesion, especially for gallbladder cancer. These histological changes suggested the characteristics of biliary carcinogenesis in PBM.

Chronic inflammation is a pathological condition associated with tissue damage and cancer. It might promote early changes in the tumor suppressor gene TP53. This seems to be the earliest and one of the most important carcinogenic pathways involved [17]. TP53 is correlated with the immunostaining of p53 (p53 protein overexpression) [18].

The present study indicated that the expression of p53 was not significantly different between the high score and low score groups. The expression of p53 appears to be related only to early epithelial changes and events leading to carcinogenesis in patients with PBM. It does not seem to play a role in indicating the pre-carcinogenic state.

Dysplasia and gallbladder cancer were observed only in the high score group. Ki67 LI was significantly higher in the high score group than in the low score group, indicating that epithelial cells had more proliferative activity in the high score group. Manuela et al. [19] reported that the investigation of Ki67 provides important information regarding the process of carcinogenesis in the gallbladder on the background of chronic cholecystitis. Therefore, the degree of inflammation might be more severe in the high score group than in the low score group. The confluent forms in the high score group might have additional effects on increased proliferation activity and subsequent malignant transformation of the gallbladder epithelium.

The present study showed the clinical significance of the confluent form in patients with PBM. The confluent form in patients with PBM requires attention. Long-term follow-up is necessary for patients with 2 or 3 elements of the predictive criteria for gallbladder carcinogenesis (cosϴ ≥0.84, Cb ≥ 8.0, and MCBD/Db ≤ 4.3) [6].

### 4.1 Limitations

Although not observed in our study, it is known that there are some PBM patients with complicated confluent forms of pancreatic and bile ducts. Such patients cannot be evaluated by the three parameters (cosϴ, Cb, and MCBD/Db) used in this study, and their histopathological progression and development cannot be predicted using our proposed predictive criteria. Hence, further studies involving patients with such complicated confluent forms are necessary.

## 5 Conclusion

In conclusion, there is a close relationship between the confluent form of PBM (evaluated using cosϴ, Cb, and MCBD/Db) and the histopathological findings of the gallbladder in PBM patients. PBM with the presence of two or three elements of the prediction criteria for gallbladder cancer (cosϴ≥0.84, Cb ≥ 8.0, and MCBD/Db ≤ 4.3) might have additional effects on increased proliferation activity and subsequent malignant transformation of the gallbladder epithelium. Long-term follow-up is necessary because such patients have a high potential for gallbladder carcinogenesis.

## Ethical approval

This study was approved by the ethics committee of Osaka City University (No.4013).

## Sources of funding

All authors declare that they have no funding source.

## Author contribution

Masaki Horiike (MH) made the conception and design of this study. Authors other than MH contributed to the collection, analysis, and interpretation of the data. MH wrote the draft manuscript, and other authors performed the critical revision of the manuscript. All authors gave final approval of the version to be published. MH has overall responsibility and guarantees the scientific integrity.

## Trial registry number

Name of the registry: Masaki Horiike
Unique Identifying number or registration ID: researchregistry5920
Hyperlink to your specific registration (must be publicly accessible and will be checked): https://www.researchregistry.com/browse-the-registry#home/registrationdetails/5f390d6bdbbef40015cf72f2/

## Guarantor

MH has overall responsibility and guarantees the scientific integrity.

## Consent

This study was conducted using surgically removed specimens, and did not target patients. Informed consent had been obtained by opt-out for research use of specimens. The opt-outs for which information is disclosed are as follows:

http://www.med.osaka-cu.ac.jp/ocucrb/doc/optout/4013.pdf.

## Availability of data and materials

Not applicable.

## Provenance and peer review

Not commissioned, externally, peer-reviewed.

## Declaration of competing interest

All authors declare that they have no conflicts of interest.
